# “A Profound, Abiding Hatred”: An Analysis of Hermann Goering’s Alleged Morphine Addiction

**DOI:** 10.7759/cureus.36865

**Published:** 2023-03-29

**Authors:** Matthew D Turner

**Affiliations:** 1 Medicine, Madigan Army Medical Center, Lakewood, USA

**Keywords:** military history, morphine use, hermann goering, medical history, substance addiction, opioid withdrawal

## Abstract

Hermann Goering, one of the most prominent members of the Nazi Party and for many years the presumed heir to Adolf Hitler, had a well-known history of morphine addiction. By the last days of the Second World War, he was widely considered by his contemporaries to have been completely incapacitated by his addiction. In this article, we argue that Goering's addiction, while possibly present, was purposefully exaggerated by his rivals for political purposes. His habit of ingesting paracodeine pills may have been a crude form of opioid maintenance therapy, similar to that of methadone today. Ultimately, his history of substance abuse had no significant impact on his capacity, ruthlessness, and leading role in Nazi crimes.

## Introduction and background

In 1945, as the remains of Nazi Germany collapsed in the last few days of the Second World War in Europe, Hermann Goering surrendered to Allied forces. The “No. 2 Nazi” [1], a man who had held “nearly unchecked power” for the past 12 years [2] brought with him 49 suitcases overflowing with looted artwork and treasures [2]. Among Goering’s possessions were two suitcases that contained over 20,000 paracodeine pills [3]. Goering had a well-known history of a debilitating morphine addiction that had forced him into involuntary commitment several decades earlier [4]. This, combined with other Nazi’s dismissal of him as a “slave” to his addiction [5], has made it easy for both his contemporaries and historians to downplay Goering as “nothing more than [a] self-indulgent, pleasure-seeking, drug-impregnated bag of lard” [6].

In this article, we argue that nothing could be further from the truth. Goering was highly intelligent, capable, ruthless, dangerous, and fully complicit in Nazi crimes. His well-documented use of paracodeine pills [3] was possibly a crude form of opioid maintenance therapy comparable to methadone today [7], and rumors of his debilitating addiction were likely exaggerated by his rivals in the endless Nazi bid for power. We argue that Hermann Goering was likely not addicted to paracodeine, and he was by no means incapacitated by it.

## Review

Hermann Goering

Speaking of her fourth-born child, Franziska Tiefenbrunn once announced, “Hermann will either be a great man or a great criminal!” [8]. History would prove the latter half of her statement correct.

Born in the German Empire of 1893 [9], Goering’s earliest memory was “bashing his mother in the face with both fists when she came to embrace him after a prolonged absence, at the age of three” [8]. Decades later, he still laughed at the memory when he told the story to Dr. G. M. Gilbert, his prison-assigned psychologist during the Nuremberg trials. This heady cocktail of “natural aggressiveness and exuberance” would define him for the rest of his life, particularly as he formed a “passion for things military and bellicose” from a very young age [8]. As an adolescent, he developed what Gilbert referred to as a “rich and vivid fantasy life which sometimes blurred the distinctions between reality and fancy” - he often had extremely powerful visions of castles coming alive with medieval characters, of Roman chariots crashing through the landscapes, and perhaps from this developed a nearly-complete “insensitivity to danger” [8]. On two separate occasions during his adolescence, he had an extremely close brush with death, once due to an oncoming avalanche and once due to a waterfall on a rowboat. Gilbert thought that Goering simply did not believe that “any harm could really befall him” [8]. Writing after the fall of the Third Reich, Gilbert concluded that this combination of traits, along with Goering’s tendency toward “aggressive egotism… domination of the environment… emotional insensitivity… [were] the seeds of outward physical boldness and moral depravity” [8].

Goering enrolled in an officer’s academy at age 16, where Gilbert states he “made his emotional transference all the more to the military authoritarian hierarchy with the Kaiser at the top” [8]. At the outbreak of the First World War in 1914, he was sent to the Western Front as a lieutenant in an infantry regiment [8]. Eager for more glory [8], he became a pilot - first on a reconnaissance aircraft taking photographs of the front (a position where he became the first German airman to carry a machine gun on a plane) - and then a fighter pilot in October 1915 [4]. He was a talented pilot, downing 28 other planes during the war [8]. Although he took a “serious wound in his hip” in a confrontation that left 60 bullet holes in his plane and forced him out of commission “for the greater part of a year” [4], he returned to action as soon as he was able. In 1918, he was given the *Pour Le Merite*, the highest military award that the German Empire could give, “not for some single action of outstanding bravery, but for continuous courage in action” [4]. Soon afterward, he was put in command of Manfred von Richthofen’s personal squadron after the famous German ace (better known as the “Red Baron”) was killed in action [4].

The First World War had given Goering the highest prestige that his nation could bestow on a military officer [4], and perhaps that is why he reacted so violently to its end. When he heard of the November 11, 1918 armistice, he furiously ordered his squadron to crash-land as many of their planes as possible to prevent them from falling into Entente hands and darkly promised his fellow officers, “Our time will come again” [4]. In the immediate chaos of postwar Germany, he discovered a talent for public speaking and announced at a meeting of former soldiers: “… I implore you to cherish hatred - a profound, abiding hatred of those animals who have outraged the German people… But the day will come when we will drive them away out of our Germany. Prepare for that day. Arm yourselves for that day. Work for that day.” [4].

Four years later, while he was studying at the University of Munich [4], Gilbert states that Goering’s “infantile emotional dependence” finally found “an authoritarian figure to cling to” [8]. Interestingly, although Goering told Gilbert, his psychologist at Nuremberg, that he joined Adolf Hitler’s burgeoning NSDAP (*Nationalsozialistische Deutsche Arbeiterpartei*, better known as the Nazi Party) out of “pure patriotic idealism” [8] and would claim that Hitler spoke “as if from my own soul” [4], his prison psychiatrist, Dr. Douglas Kelley, came to a different conclusion. Goering told Kelley in an interview that he joined the Nazi Party because, “… I wanted to help destroy the [Weimar] Republic and to be, perhaps, the ruler of the new Reich” [3]. He claimed that he was not attracted to the party because of Hitler in particular, but because he saw promise in the small party, where “I could soon be a big man in it” [3]. In fact, he had previously tried to form a “revolutionary party” himself but found that he had no knack for it [8], suggesting that he joined the Nazi Party largely for his own self-advancement. These two alternating viewpoints are the fundamental dichotomy of Hermann Goering - on one hand, we have a caricature of a hapless sycophant groveling at Hitler’s feet nicknamed “Fatty Hermann” [6], a yes-man who “early learned to escape in fantasy, illness or drug addiction” [8]. On the other hand, we have a “brilliant, brave, ruthless, grasping, shrewd man”- a highly intelligent individual capable of long-term planning and “cold-blooded political pragmatism” [3].

For his part, Hitler was thrilled to have the famous war hero join the Nazi ranks. Almost immediately, Goering was placed in the highest levels of the Party, and put in command of the *Sturmabteilung* (SA), a “semi-military, voluntary organization of young men trained for and committed to the use of violence, whose mission was to make the Party the master of the streets” [10]. Goering would later brag that he turned the unruly SA from a mob into an organized fighting force [4]. Hitler often praised him in the early days, stating that, “I liked him… I gave him a disheveled rabble. In a very short time he had organized a division of eleven thousand men” [4].

By late 1923, Hitler was growing impatient. Certain that the local Bavarian government was weak and vulnerable, the Nazi Party attempted a “quick, careless, and melodramatic” coup at the *Bürgerbräukeller* Beer Hall in Munich [4]. With Goering, the rest of the Nazi leadership, and 3,000 SA stormtroopers, Hitler marched on Munich’s War Ministry on November 8, 1923 [4]. Violence soon broke out when the stormtroopers and local police exchanged fire, and in the altercation, Goering, marching at the head of the mob, was shot. Accounts differ on the exact placement of his wound - some sources claim he sustained “a bullet in the upper thigh” of his right leg [3,11], while others claim that he was “badly shot in the groin” [4], and one even claims that he suffered a “stomach injury” instead [12]. As chaos broke out in the streets and blood poured out of Goering’s wound, he was dragged into a furniture shop where the Jewish owner’s wife helped treat his wounds [4]. From there, the Nazis smuggled him to the clinic of a friend named Professor Alwin Ritter von Ach, where his wound was further cleaned and dressed [11]. Several days later, Goering and his wife slipped across the border into Austria [11]. Hitler was arrested on 11 November [4] and not released from prison until December 20, 1924 - it is notable that even after Hitler’s release, the two men would not meet again for several years “until Hitler’s star was once more in the ascendant” [8].

Three weeks after his injury, Goering, on the run and with little resources to his name, still had not received proper care. His wife finally took him to an Innsbruck Hospital, where he underwent multiple operations to remove bullet fragments, “dirt from the street buried in his thigh muscles” and to drain the “horrible amount of pus” present [4]. Writing to her mother on 30 November, she noted: “Hermann is in a terrible state. His leg hurts so much he can hardly bear it… All the time he is suffering indescribable pain…” [4]. It was at this time that his physicians prescribed “two injections a day” of morphine for his pain and fever [4]. By 8 December, his condition had still not improved: “… He bites the pillow because it hurts him so much, and he moans all the time… in spite of being dosed with morphine every day, his pain stays as bad as ever” [4].

Goering was finally discharged from the Innsbruck Hospital on 24 December. By this time, he seems to have developed a dependency on morphine; for when he fled to Italy soon afterward, he was noted to have “kept himself going with morphine” [4]. During this time, his wound did not heal properly, and he walked with a noticeable limp. From 1924 through 1925, Goering “lost his youthful physique and began to develop the girth that characterized him later on” [13]. By 1925, his physician was shocked to find that the former war hero had “a body like an elderly woman, fat, pale, and white” [4]. Goering injected himself with morphine on a daily basis, and by that summer, had developed “the worst symptoms”, with occasional “outbreaks of violence” [4]. He “looked awful… the trim and handsome young officer of 1918 had turned into a pale, puffy-faced invalid. Daily drug injections had made him listless… the combination of inactivity and overeating added more than forty pounds to his physique” [14]. In the fall of that year, while under observation in a hospital, he violently attacked a nurse who refused to give him morphine. Following this, he was committed to the Långbro Institute for the Cure of Nervous Diseases in Sweden [4] and confined to a padded cell for three days [14]. His first stay at Långbro lasted three months and required the use of a straitjacket [4]. Although some sources state that this initial visit was enough for Goering “to divest himself of his morphine addiction” by 1926 [3,13], with his physician even granting him a “certificate of sanity” on October 7, 1925 [14], this appears to have not been the case. Goering had to return to Långbro several times to treat his addiction, with his last visit in 1927. By this point, he appears to have been largely, if not completely, cured of his morphine addiction, evidenced by his improving relationships with his family [15] and by re-entering the world of Nazi politics when President von Hindenburg gave him an amnesty for his part in the Beer Hall Putsch [4]. During the Nuremberg Trials decades later, Goering would admit to his psychiatrist that he took small doses of morphine for a “very severe sore throat” two years later in 1929 but denied that this had been tied to his previous addiction [3].

When it was safe for him to return to Germany, Goering quickly re-established contact with Hitler. Winning a seat in the Reichstag in 1928, he established himself as the pleasant, public face of the Nazi party, and was known both as the “salon Nazi” and the “Ambassador of Hitler” [4]. Although Hitler initially regarded him coolly - perhaps because of Goering’s noted absence from Germany for the past several years - by 1931, he was back on friendly terms with Goering, gifting him a large Mercedes for his political work [4]. The very next year, as the Nazi Party’s political power grew at an exponential rate, Goering was elected President of the Reichstag, an incredible “triumph for the man… who only seven years before had been confined to a strait-jacket” [4]. Hitler came to speak of his “first political assistant” [1] glowingly, writing: “After receiving a grave wound you again entered the ranks as soon as circumstances permitted as my most loyal comrade for power. You contributed essentially to creating the basis for the 30th of January” [1].

When writing of the 30th of January, Hitler was referring to his inauguration as the Chancellor of Germany on January 30, 1933. Less than a month later, he used the opportunity granted by the Reichstag Fire to vastly expand his powers over Germany [4]. Goering was one of the first on the scene at the Reichstag Fire, and there is controversy to this day as to whether or not he played a role in setting the blaze that swept the Nazis into absolute control of the Third Reich [4]. Whatever the cause of the fire, Goering’s place in the “inner circles of Nazi power” [16] allowed him to rapidly accumulate a vast array of offices. As Prussian Minister of the Interior, he “proceeded promptly to establish a regime of terror in Prussia designed to suppress all opposition to the Nazi program” [1] and founded the *Die Geheime Staatspolizei* (Secret State Police) that would become the Gestapo [10]. In a few short years, he was not only *Reichsmarschall* of the *Luftwaffe *(Germany’s air force), the Successor Designate to Hitler, but also “under Hitler, the Chief Executive of the Nazi state”, placed in charge of the 1936 Four Year Plan meant to put the German economy on a war footing, and was widely recognized as the “No. 2 Nazi… a leading participant in every major plan of territorial aggrandizement or offensive military strategy” [1]. With such “extraordinary economic powers” at his fingertips [9], the “second man in the Reich’s” [9] health began to deteriorate. The period of 1937-1938, as the specter of war grew on the horizon, was especially difficult. His weight, still not recovered from the 1920s, often rose “well above the 280 pounds that he considered to be his limit” as the “busiest man in the Reich… in charge of squeezing blood out of stones” frantically worked 18-hour days [17] as he struggled to make the Third Reich economically independent of the neighbors it would soon invade [12]. His job became exponentially more difficult when he learned that Hitler had moved the date for war from 1942 to 1939 [14]. Mosley describes Goering’s behavior at this time as an ever-repeating “crisis of overwork [leading to] the next bout of overindulgence” [17].

It was in this frenetic atmosphere that disaster struck. In the frantic days of 1937, Goering received an important phone call from Hitler while he was in the sauna. Eager to not disappoint his *Führer*, he spent two hours discussing the upcoming annexation of Austria with Hitler, sitting “in a drafty corner of [a] gymnasium with no covering other than a towel around his waist” [17]. The next day, he experienced a fever and a rapidly worsening toothache. He reached out to his dentist, American-trained professor Hugo Blaschke, and was prescribed a short course of paracodeine pills - a recently developed mild morphine derivative [17]. Five days later, Goering called Blaschke, demanding more of the pills. Blaschke refused to prescribe more, but Goering soon found his own source. By the end of the year, “he was taking about ten of the pills a day” [17].

Goering’s paracodeine pills were a habit that he would continue until his capture by Allied forces in 1945 [3], so much so that one American colonel said of meeting him: “When Goering came to me at Montfort, he was a simpering slob with two suitcases full of paracodeine. I thought he was a drug salesman” [17]. According to Kelley, at the time of Goering’s capture, he possessed the entire German stock of paracodeine - and since paracodeine was not produced outside Germany’s borders, he thus owned “the entire world supply” [3]. One source estimated he had over “20,000 paracodeine pills” at the time of his capture [18].

Although he remained a central figure in “every phase of the Nazi conspiracy” [1], Goering’s star faded as the Second World War dragged on. His crimes - which included the massive use of slave labor and human experimentation on behalf of the Luftwaffe, the widespread looting of Europe’s finest works of art, the plundering of vast regions of conquered land, a key player in the Holocaust, and a pro-war voice for continued German aggression [1] - are far too numerous to be adequately addressed in this article. The Luftwaffe’s mounting failures at Dunkirk, the Battle of Britain, and the disastrous attempted airlift of supplies to the trapped Sixth Army at Stalingrad significantly hurt his reputation [4]. In one particularly embarrassing episode, he arrogantly declared in September 1939, “No enemy bomber can reach the Ruhr. If one reaches the Ruhr, my name is not Hermann Goering. You may call me Hermann Meyer.” The British Royal Air Force began bombing German cities less than a year later [4]. As the war dragged on, Hitler began to lose patience with Goering, often screaming about “the inefficiency and uselessness of the Luftwaffe with such contempt and viciousness…” [8]. Retreating back into his childhood “protective fantasies,” as Gilbert describes them, Goering often promised Hitler secret “wonder-weapons” that never materialized, did his best to hide crucial information from the *Führer*, and came to be ignored by other high-ranking Nazis as his power diminished [8]. Eventually, “if Goering’s name was so much as mentioned, Hitler would start fuming at the mistakes and omissions in the planning for air warfare” [19]. By the spring of 1945, Albert Speer recalled that Hitler’s dislike and mistrust of Goering had grown to the point where had no qualms about publicly insulting the Reich Marshal “in the most cutting manner” [19].

The final end to Goering’s career came in April 1945. As the Soviet army advanced into Berlin, Hitler announced that he was planning to commit suicide rather than surrender the city. Upon hearing this, Goering immediately telegrammed the *Führerbunker *where the last remnants of Hitler’s entourage hid from the Battle of Berlin. Remembering the days when he had been regarded as the presumptive heir, Goering asked for permission to assume leadership of Germany in the event of Hitler’s death [4]. Unfortunately for Goering, Hitler’s personal secretary, Martin Bormann, intercepted the telegram and with the aid of Joseph Goebbels, the Nazi chief of propaganda, informed Hitler that this was likely a coup attempt [4]. Furious at this perceived betrayal, Hitler stripped Goering of his rank, exiled him from the ranks of the Nazi Party, and sent the Schutzstaffel Nazi paramilitary organization (SS) a warrant for his arrest [4]. Goering only narrowly evaded execution and was soon captured by American forces [8].

The “last act of the Goering drama” ended with his incarceration and trial at Nuremberg [8]. Presenting a “front of utter amiability and good-humored bravado” [8], he displayed absolutely no remorse for his actions and confessed to Kelly a “single driving ambition - to achieve for Hermann Goering supreme command over the Third Reich” [3]. At Nuremberg, he achieved his wish, swiftly bullying his way into a leadership position over the other Nazi prisoners [3]. His sharp wit, extremely inappropriate use of humor at the trials [20], and tendency to make others underestimate him [6] initially made Goering a formidable opponent for the prosecution, and he was regarded by Willis Smith, president of the American Bar Association, as “the most versatile and able of the group” [21]. He was noted to “dominate the proceedings” with “immense ability and knowledge” and was regarded as “suave, shrewd, adroit, capable, [and] resourceful” by the prosecution [4]. Driven by his ego to the end, “everything he said and did during the trial… was designed to build himself up for posterity” [3], and he once boasted to Kelley, “In 50 or 60 years there will be statues of Hermann Goering all over Germany, little statues maybe, but one in every German house” [3].

Almost 80 years later, no such statues exist. For all his bluster, Goering’s arguments and defenses collapsed in the face of such overwhelming evidence. “At Nuremberg he was a *Führer *without a country, a marshal without an army, a prisoner accused of wagging aggressive war against peaceful people and of the deliberate murder of millions” [3]. In 1946, the International Military Tribunal found him guilty of all four counts with which he was charged: conspiracy, waging a war of aggression, war crimes, and crimes against humanity [22]. The court’s final verdict was damning in its completeness: “There is nothing to be said in mitigation. For Goering was often, indeed almost always, the moving force, second only to his leader… his own admissions are more than sufficiently wide to be conclusive of his guilt. His guilt is unique in its enormity. The record discloses no excuses for this man” [22]. The night before his scheduled death by hanging, Goering committed suicide via cyanide pill [4]. For years afterward, rumors circulated that his body was incinerated in one of the ovens at the Dachau concentration camp meant for prisoners [23]. Along with the remains of 10 other high-ranking Nazis, his ashes were poured into the River Isar, and from there to the sea. Like the others, Goering “had sought to achieve greatness in history… they built upon pillars of hate, and what they stood for could not stand” [23].

Goering’s possible addiction at the time of his capture

From the information gathered above, it is certain that Goering used morphine or morphine derivatives for significant periods of his adult life. From 1937 to his capture in 1945, his daily use of paracodeine pills [17] made it “dangerously possible to assume that he was… nothing more than [a] self-indulgent, pleasure-seeking, drug-impregnated bag of lard with whom Hitler had lost patience” [6]. As the war progressed and his influence waned, other Nazis seem to have dismissed Goering as little more than a drug addict. One SS commander stated to Gilbert that Goering’s “drug addiction and corruption” made him write off Goering as any sort of “moderating” influence on Hitler’s increasingly erratic behavior, while one of Joseph Goebbels' aides claimed that Dr. Theodor Morell, Hitler’s personal physician, had said that Goering “was becoming more and more a slave to the habit” [5]. In his book *Blitzed: Drugs in the Third Reich*, Ohler paints a picture of Goering injecting massive amounts of morphine directly into his veins, directly leading to an ill-fated meeting with Hitler that resulted in the irrational decision to halt the German armies outside of the trapped British forces at Dunkirk [12]. Similarly, he describes how Goering would often leave a room “without any explanation” when “the opium content of his blood had dropped”, then return, visibly refreshed from a fresh dose [12].

However, it is important to note that many of these recollections are directly tied to other prominent members of the Nazi Party. Far from being a united front behind Hitler, the National Socialist government was a vicious quagmire of rival groups and individuals endlessly jockeying for the power that proximity to the *Führer *gave - a dangerous prospect in its own right, given Hitler’s unpredictable whims. This “polycratic governing system of rivalry, duplication, patronage and self-promotion” made for an environment of endless self-aggrandizement and backstabbing in a “regime [that] was shot through by inefficiency, corruption, and self-interest” [24]. Goering was an integral part of this world, and a common target for jealous scorn: an SA leader once furiously swore he’d “blow that pig Goering’s brains out” [14]. Goering was more than willing to strike down others as well. In 1934, he ordered a close friend and ally, Ernst Röhm, shot in a power struggle within the Nazi Party. Years later, when asked about this murder, he shrugged and simply said, “He was in my way” [3]. As his status as the “No. 2 Nazi” [1] declined, Goering’s political dealings became more desperate. At one point during the war, Goering conspired with several others to “form a solid fence” around Hitler to limit his rival’s influence [19]. In this regard, he was outmaneuvered by Bormann, who used his unique position as Hitler’s personal secretary to reduce Goering’s status in the halls of power as early as the spring of 1943 [19] and played a key role in persuading Hitler to order Goering’s arrest and execution in April 1945 [4]. In such a backstabbing, politically unstable environment - the same sort of environment that Goering had used to help ruthlessly purge Röhm and the very same SA he had once led in the 1934 “Night of the Long Knives” [4] - emphasizing and inflating tales of Goering’s drug addiction would have served his rivals well. One of the main sources that Ohler uses to describe Goering’s drug addiction is Albert Speer’s memoir *Inside the Third Reich* [12]. Speer, a noted architect and the Nazi Minister of Armaments and War Production, is a doubtful source at best - his memoirs are riddled with contradictions, particularly when he attempts to downplay his role in the Holocaust and cast himself in a more sympathetic light [25]. During the Nuremberg Trials, Speer was a constant thorn in Goering’s side. On one occasion, he undermined Goering’s attempt to have the prisoners present a “united front” by accusing him of plotting to assassinate Hitler in February 1945. Goering furiously denied the charge, but the damage was done, foiling his plans [26]. Perhaps to further damage his rival and make himself appear better in comparison, Speer also scornfully accused Goering during the Nuremberg Trials of “doping himself up with morphine and stealing art treasures from all over Europe when Germany was in agony” [8]. Even the charge made by Goebbels’ aide mentioned above - remembering a comment that he had supposedly heard in passing - is suspicious, given that Goebbels was another key rival in Goering’s bid for power, and also played a significant role in Hitler’s decision to oust Goering from the party [4]. The SS officer’s report may be viewed in a similar vein. In 1942, Reinhard Heydrich, the head of the Reich Security Main Office, and possibly working under the influence of Heinrich Himmler, the head of the SS, proposed a law that called for drug addicts to be included on the list of “anti-socials” within the Reich. The cabinet, which included Goering at the time, quickly struck down the proposal, for the bill would have “vastly expanded the policing power of the SS” [5]. After such a rebuff, it would have been in the SS’s interest to portray Goering as an incapacitated drug addict. Goering himself later grumbled at Nuremberg that he would have shot Himmler himself rather than give him over to the Allies [26].

Ultimately, it appears that many of these rumors may have been deliberate falsifications or exaggerations meant to undermine Goering’s status in the upper ranks of the Nazi Party. As Speer himself admits in* Inside the Third Reich*: “Goering was an easy mark…” [19]. By the last days of the war, Goering had been politically crippled by his rivals. “His decline, once it had begun, was hastened by the rivalry and envy of other top Nazis, and he was virtually in retirement during the last few months of fighting” [3].

Goering himself was aware that his history of morphine addiction was a potential political liability and had his medical records from Långbro destroyed in the early 1930s. However, by this point, the secret was already so well-known that even the foreign diplomatic corps was aware of his history [5]. “Well aware of the dangers of overindulgence,” Goering was “determined that never again would he fall victim to the addiction which had brought him low in Sweden” and was careful to monitor his intake of paracodeine beginning in 1937 [17]. In addition to this, approximately once a year, he would visit the clinic of Professor Hubert Kahle in Cologne to help aid in any withdrawal symptoms, typically in the form of sleeping pills. He believed that this, combined with a sauna at his private mansion, helped control his addiction [5]. Despite his unhealthy weight - nearly 280 pounds at the time of his 1945 capture - Goering also displayed “meticulous attention” to his body [3], though whether this was out of narcissism [3] or a desire to avoid repeating his past negative experiences is impossible to determine.

Upon interviewing Goering at Nuremberg, Kelley was doubtful that the Nazi was even truly addicted to his paracodeine pills. Kelley maintained that Goering “had the paracodeine pill habit, in the way that many people have the cigarette habit. It was the need to do something with his hands and mouth, to perform an act he was accustomed to, and liked doing” [3]. From 1937 to 1945, Goering would typically have a bottle on his desk that contained 100 or so of the paracodeine pills, often “chew[ing] them leisurely” during conferences and conversation [3]. This “habit”, Kelley maintains, offered Goering no particular stimulation [3]. It could be compared to Goering’s habit of “always nibbling” on snacks in the stressful years of 1937-1938 [17]. Kelley further stated that each of the pills “contained only a small amount of paracodeine” - by the time of his capture in 1945, Goering took approximately 100 daily, “not an unusually large dose… not enough to have affected his mental processes at any time” [3].

Kelley estimated that Goering’s daily dose of 100 paracodeine pills in 1945 was equivalent to 3-4 grains of morphine daily [3]. This obsolete unit of measurement translates to approximately 64.8 milligrams (mg) of morphine per grain [27]. Given this, by Kelley’s estimate, Goering was taking 194.4 mg to 259.2 mg of morphine per day. In comparison, a 2015 study of patients with therapeutic opioid addiction (TOA) in response to chronic noncancer pain had a daily mean dose of 215.93 mg (±231.44) of morphine. Those without TOA had a daily mean dose of 71.11 mg (±71.65) of morphine [28]. The researchers concluded that opioid dosage was a “moderately accurate predictor” of TOA [28]. As Goering had a previous history of substance abuse in the 1920s [4], an optimal cut-point of 117.50 mg of morphine daily has a positive predictive value of 100% and a negative predictive value of 28.20% for TOA [28]. Given that Goering’s daily dose could have easily been more than twice that [3], and that daily average oral morphine equivalence is “strongly associated with increased odds of TOA” [28], it appears that Kelley’s assertion that Goering was taking “not an unusually large dose” [3] is incorrect. While Goering weighed approximately 280 pounds at this time, studies have shown that patient weight is not significantly associated with the degree of analgesia that morphine provides [29]. His self-confessed obesity [3] would not have had a protective effect against the dosage of morphine he was ingesting. These data strongly suggest that *Reichsmarschall’*s “habit” [3] was a fully-fledged addiction by 1945.

However, these data are also suspect - while Kelley reported Goering took approximately 100 pills a day, his original captor, Colonel Andrus at Ashcan Prison, reported earlier that Goering “has been in the habit of taking 20 pills per dose, two doses a day” [5]. Andrus’ estimation, which is only 40% that of Kelley’s, puts Goering at a much lower daily dose of morphine. It is possible that both men’s accounts are accurate. Andrus did nothing to wean Goering off the paracodeine pills, and in the stressful situation of being captured, the Nazi may have simply increased the nervous habit that offered him a degree of comfort, increasing his daily dose to 100 pills by the time he came into Kelley’s care [3]. In 1937, Goering was reported to have taken only 10 of the pills per day [17], roughly equivalent by our estimates to 19.4 mg to 25.9 mg of morphine daily. If Andrus is correct, then Goering’s consumption quadrupled over the course of the next eight years, a remarkably slow progression if he were truly addicted. In addition to this, paracodeine was only produced within Germany at the time [3], a fact which throws Kelley’s estimation of the morphine equivalence of Goering’s pills into doubt - particularly since only 5%-15% of dihydrocodeine, the active agent in paracodeine, metabolizes into the active form [30].

Some researchers have suggested that Kelley deliberately downplayed Goering’s addiction due to “the American justice system and its requirement that the condemned must appear sound of body and mind before going to the gallows… it is not conclusive that Goering was cured at all” [5]. Leon Goldensohn, the psychiatrist who eventually took Kelley’s place, theorized that Goering’s “drug addiction is far from cured… he is still a basically weak character resorting to other means of frustration-evasion and ego-protecting” [5]. Indeed, Kelley and Goering seem to have become inappropriately close; at one point Goering asked Kelley to adopt his daughter Edda if his wife were to die [2]. Kelley’s suicide via cyanide capsule in 1958 also uncannily mirrors Goering’s end [2]. While Kelley’s bias toward Goering is certainly possible, the features that Goldensohn noted and the “domineering aggressiveness” [8] that Goering displayed in the last months of his life can more likely be attributed to his status as an “aggressive psychopath” [20] and his desperate desire to secure a lasting legacy for himself [8] than any withdrawal symptoms. Even Goering’s suicide was a calculated act of defiance rather than despair, an attempt to achieve martyrdom and “to make a mockery of human values and to distract attention from his guilt by a dramatic gesture” [20].

While Goering’s high daily dose (40 or 100 paracodeine pills daily, depending on the source) in 1945 implies a morphine addiction, Kelley reports that it was remarkably easy to wean Goering off his paracodeine pills, simply by “cutting down the dosage each day until no more drug was allowed” [3]. Goering endured this stoically, only complaining of occasional pains which were easily relieved “by other sedatives” [3]. Kelley found that appealing to Goering’s narcissistic ego aided this process significantly [3]. Other than the “occasional pains” that Kelley recorded - most of which he had to draw out of Goering, who was reluctant to discuss his discomfort [3] - Goering did not display any other withdrawal symptoms. Typically opioid withdrawal symptoms (OWS) involve a range of unpleasant symptoms including agitation, anxiety, muscle aches, insomnia, tachycardia, hypertension, dilated pupils, anorexia, nausea, diarrhea, and vomiting [31]. There are no records of this - if anything Goering was remarkably clear-headed and intelligent throughout the trial. Diet and exercise rapidly improved his health, and by October 1945, his “health and confidence” had visibly improved [18]. Much to his pride, he scored an IQ of 138 on a German version of the American Wechsler-Bellevue Adult Intelligence Test, placing him among the three most intelligent of the 21 Nazis tested [17]. Even out of the acute phase of OWS, patients may experience “irritability, decreased ability to focus, and deficits in executive control that can last for months” [32]. It is difficult to imagine that a man in the throes of acute and chronic morphine withdrawal would be able to perform so capably in both academic tests and his defense [4].

The “humane treatment” that Goering received during his incarceration by Allied forces [21] was a great boon for his health. When captured, he weighed nearly 280 pounds, and “even he was willing to admit that he was somewhat bulgy” [3]. Five months later, he was down to 200 pounds [3]. Part of this was due to his diet - oatmeal and bread for breakfast, soup, fish, and salad for lunch, and bean stew and bread for dinner [18] - but a large portion of this was also due to his fierce desire to “make a better appearance in court” [3]. By the time of his trial, “his trousers were baggy because prison food and some exercise had made him fitter than he had been for years” [6]. His beloved uniform became baggy and ill-fitting [18], and many onlookers were shocked at how different the 53-year-old looked from the pictures “that showed him in his heyday of power” [21]. This clinical picture does not correlate with a man dealing with the sudden discontinuation of decades of morphine addiction [32].

Goering’s morphine dependency

From this evidence, we suggest that Goering did not suffer from an opioid addiction at the time of his capture, but rather an opioid dependency. Although Ohler states that when captured, Goering possessed thousands of pills of Eukodal (an early form of oxycodone) [12], Kelley is very clear that Goering’s opioids came in the form of paracodeine. Better known today as dihydrocodeine (DHC), this drug was invented in Germany in 1908 and released for public consumption in 1911 [33]. At the time of Goering’s capture, DHC was not produced anywhere outside of Germany [3], and thus, it is highly unlikely that Kelley or any of the other Allied physicians in charge of Goering’s care were aware of the full range of its effects.

DHC is one of the weaker agents used to treat pain and is similar in efficacy to tramadol. With a short half-life of only several hours, it only has a weak binding affinity for the mu-opioid receptor and is most effective in the form of dihydromorphine, one of its metabolites [34]. During the body’s metabolism of DHC, only 5%-15% of the drug is converted into this active form of morphine [30]. Remarkably, a 1998 German study found that treating opiate addicts with maintenance doses of DHC was comparable to maintenance treatment with methadone [30]. This finding has since been replicated by other researchers, providing evidence that “dihydrocodeine is a viable alternative to methadone as a maintenance treatment for opiate dependency” [34]. In a similar vein, maintenance DHC therapy may also be used to treat patients with severe alcoholism [35].

A 2020 systematic review of DHC’s potential for opioid maintenance therapy examined three separate trials in five articles and cautiously concluded that DHC may be comparable to methadone for the maintenance treatment of opioid addiction. While the researchers admitted the review was undermined by lower-quality evidence [7], the possibility remains intriguing.

We propose the following theory: in 1937, during the busiest days of his career [4], Goering, a man with a history of a severe morphine addiction throughout the 1920s [4], possibly developed opioid craving secondary to the extraordinary amounts of stress his mind and body were undergoing [36]. At the time, his food consumption appears consistent with binging and purging episodes [17], possibly implying an overlap between his excessive over-eating and history of substance use disorder [37]. Even if he had tried to get morphine from Berlin pharmacists, it would have been extraordinarily difficult, as the medical community was well aware of his history of substance abuse [38]. However, when he was prescribed paracodeine for his toothache in 1937 [17], Goering may have inadvertently stumbled across a maintenance treatment for his opioid cravings, similar to that of methadone. DHC has a much shorter half-life than methadone and, for maintenance treatment, has to be administered several times a day [7], consistent with Kelley’s description of how Goering dosed himself [3]. Over the years, Goering received little in the way of “stimulation” from the pills [3]. Well aware of the devastating effects of his morphine addiction in the 1920s, and terrified of the political implications such an addiction could bring him [17], Goering may have subconsciously kept dosing himself with paracodeine to keep his opioid cravings at bay as his status within the Nazi Party faded. When he was eventually weaned off the pills, he displayed little, if any, of the expected side effects of opioid withdrawal [3]. Ultimately, we suggest that Goering’s paracodeine pills were a dependency, not an addiction - they were a means of keeping his true addiction at bay even as defeat loomed and his world collapsed around him.

The current literature on addiction medicine supports this claim. Medication-assisted treatment for opiate dependency is significantly associated with better outcomes, although the data on periods for longer than one year are still lacking [39]. A 2015 review article in the Harvard Review of Psychiatry estimated that “medication-assisted treatment of opioid use disorder… at least doubles rates of opioid-abstinence outcomes” [40]. Although DHC was not included in this study, as it has not been approved by the FDA to treat addiction [40], it has a weak binding affinity for the opioid receptor [34], similar to buprenorphine. Buprenorphine has a much higher rate of medication compliance than other treatments such as Naltrexone due to this partial affinity [41]. This may explain why Goering displayed such an affinity and compliance for taking his paracodeine pills daily [3]. Research has shown that DHC can also be used to effectively treat neuropathic pain [42]. In this regard, Goering’s complaint of “occasional pains” after Kelley discontinued his paracodeine pills [3] may have been due to a re-emergence of an underlying neuropathy due to his poor health and diet, rather than a symptom of withdrawal.

Even if this theory is incorrect, it is our opinion that it is extremely dangerous and foolhardy to simply write off Hermann Goering as a drug addict. Not only does this provide a potential excuse for his reprehensible actions before and during the war [4], but it plays into Goering’s own strategy. He often embraced the role of the “regular guy” [14], an affable man of “childish enthusiasm” capable of charming even his physicians at Nuremberg [2] - leaning into his portrayal as a harmless drug addict as his political star faded and the situation became more dangerous would not have been out of character for him. And yet, far from being the incompetent, drug-addled buffoon that Bormann, Goebbels, Speer, and in time, even Hitler, portrayed him as [4], he was “nobody’s fool, not even Hitler’s” [3]. Kelley concluded that, in his place at “the nucleus of the Nazi Party… Hermann Goering performed the duties of every one of his offices effectively” [3]. It is telling that Goering was “the last man standing” of the topmost echelon of Nazis [2], and the one most determined to salvage his legacy for posterity [3]. In the end, perhaps he defeated his political rivals after all.

## Conclusions

Hermann Goering is a complex figure. A war criminal and violent psychopath, he nonetheless had a powerful charisma capable of charming even his captors. We theorize that he appears to have stumbled across a means of treating his opioid cravings through the use of paracodeine pills. His capable and intelligent behavior during the trial and his lack of withdrawal symptoms suggest that stories of his morphine addiction during the Second World War were massively overexaggerated, due to both the machinations of his rivals within the ranks of the Nazi Party and his growing political irrelevancy as the Luftwaffe’sdefeats mounted. Ultimately, we conclude that Goering’s dependence on his paracodeine pills actually helped him ward off the cravings for stronger opioids, allowing him to be a fully capable actor in Nazi crimes. He was highly capable, intelligent, and fully complicit in the horrors of the Third Reich. Attributing any of his actions and behavior to morphine addiction is a dangerous oversimplification of one of the most ruthless and dangerous psychopaths of the 20th century.
